# Report of Deaths in the City of Buffalo for the Month of June, 1863

**Published:** 1863-08

**Authors:** Sandford Eastman

**Affiliations:** Health Physician


*Report of Deaths in the City of Buffalo for the month of June,* 1863.

Accident, 4; do. by drowning, 7; Brain, concussion of, 1; Cancer of the stomach, 1; Cholera morbus, 1; Consumption, 16; Convulsions, 15; Croup, 1; Debility, 1; Diarrhœa, 2; Disease of the brain, 1; Disease of the heart, 4; Disease of the spine, 1; Diphtheria, 1; Dropsy, general, 4; do. abdominal, 2; Dysentery, 1; Fever, 1; Fever, puerperal, 1; do. scarlet, 4; Hæmorrhage, 1; do. from uterus, 2; Inflammation of the bowels, 2; do. brain, 3; do. of meninges, 5; do. lungs, 14; do. pericardium, 1; do. peritoneum, 2; do. stomach, 1; Jaundice, 2; Marasmus, 2; Murder, 2; Old age, 6; Paralysis, 1; Rheumatism, 1; Scrofula, 1; Unknown, 5; Whooping cough, 1. Total deaths from diseases, 122. Also, 3 still-born were reported.

*By whom Certified.*—By Regular Practitioners at Public Institutions, 16; do. in the City at large, 46. By Irregular Practitioners, 24; by Coroner, 17; by Undertakers, 22. Total, 125.

The following shows the number of deaths in the City in each month of the present year; the number in 1862; and the average of each month for the five years, 1858 to 1862, inclusive:

| | 1863. | 1862. | 5 years Average. |
|---|---|---|---|
| January, | 113 | 128 | 135 |
| February, | 90 | 132 | 120 |
| March, | 94 | 137 | 136 |
| April, | 119 | 155 | 121 |
| May, | 129 | 124 | 116 |
| June, | 122 | 118 | 112 |

The number of deaths in the first six months of the present year is 97 less than in the corresponding period of last year, and 73 less than the average for five years.

SANDFORD EASTMAN, M. D., Health Physician.

